# Association between body fat and sarcopenia in older adults with type 2 diabetes mellitus: A cross-sectional study

**DOI:** 10.3389/fendo.2023.1094075

**Published:** 2023-01-27

**Authors:** Lina Sun, Junling Fu, Zhijing Mu, Xiaoye Duan, Piu Chan, Shuangling Xiu

**Affiliations:** ^1^ Department of Endocrinology, Beijing Institute of Geriatrics, Xuanwu Hospital, Capital Medical University, Beijing, China; ^2^ National Clinical Research Center for Geriatric Disorders, Xuanwu Hospital, Capital Medical University, Beijing, China; ^3^ Department of Neurobiology, Neurology and Geriatrics, Beijing Institute of Geriatrics, Xuanwu Hospital of Capital Medical University, Beijing, China; ^4^ Advanced Innovation Center for Human Brain Protection, Capital Medical University, Beijing, China; ^5^ Key Laboratory for Neurodegenerative Disease of the Ministry of Education, Clinical Center for Parkinson’s Disease, Capital Medical University, Beijing, China; ^6^ Beijing Key Laboratory for Parkinson’s Disease, Xuanwu Hospital, Capital Medical University, Beijing, China; ^7^ Beijing Institute of Brain Disorders, Capital Medical University, Beijing, China

**Keywords:** body fat, sarcopenia, body composition, obesity, diabetes, China

## Abstract

**Objectives:**

To investigate the association between body fat (BF%) and sarcopenia in older adults with type 2 diabetes mellitus (T2DM) and potential link with increased levels of inflammatory indicators and insulin resistance.

**Methods:**

A total of 543 older adults with T2DM were included in this cross-sectional study. Appendicular skeletal muscle (ASM), handgrip strength and gait speed were measured to diagnose sarcopenia according to the updated Asian Working Group for Sarcopenia (AWGS) 2019 criteria. Body composition data were tested using dual-energy X-ray absorptiometry (DEXA). Levels of serum high-sensitive C-reactive protein (hs-CRP), interleukin-6, fasting blood insulin (FINS), hemoglobin A1c (HbA1c), 25-hydroxyvitamin D_3_ [25(OH) D_3_] were also determined.

**Results:**

The prevalence of sarcopenia in all participants was 8.84%, of which 11.90% were male and 5.84% females. The Pearson’s correlation analysis revealed that BF% was negatively correlated with gait speed in men and women (R =-0.195, *P*=0.001; R = -0.136, *P* =0.025, respectively). After adjusting for all potential confounders, sarcopenia was positive associated with BF% (male, OR: 1.38, 95% CI: 1.15–1.65, *P*< 0.001; female, OR: 1.30, 95% CI: 1.07–1.56, *P*=0.007), and negatively associated with body mass index (BMI) (male, OR: 0.57, 95% CI: 0.44–0.73, *P*<0.001; female, OR: 0.48, 95% CI: 0.33–0.70, *P*<0.001). No significant differences were found in hs-CRP, interleukin-6, and insulin resistance between older T2DM adults with and without sarcopenia.

**Conclusion:**

Higher BF% was linked to an increased risk of sarcopenia in older adults with T2DM, suggesting the importance of assessing BF% rather than BMI alone to manage sarcopenia.

## Introduction

Sarcopenia and obesity are two highly prevalent conditions in older adults related to body composition and are known to be associated with poor physical function (1, 2). Aging leads to increased fat mass along with loss of muscle mass and strength, even in older adults with stable body weight (3). Sarcopenia is defined as a loss of muscle mass, decline in muscle strength and/or physical function that is associated with increased risks of falls, fractures (4), physical disability, morbidity (5), and even mortality (6). Sarcopenic obesity (SO), a disorder characterized by the coexistence of obesity and sarcopenia, is associated with a higher risk of adverse outcomes than individuals with sarcopenia or obesity alone (7). To prevent the adverse outcomes of sarcopenia and/or SO, it is important to understand the impact of obesity on sarcopenia and the underlying mechanisms. An excessive amount of adipose tissue may lead to decline of muscle mass and function through oxidative stress, inflammation, and insulin resistance, suggesting that fat mass might play a role in the onset and development of sarcopenia (8).

Type 2 diabetes mellitus (T2DM) is another prevalent disease in the elderly population, affecting approximately 25% of people aged over 65 years (9). Previous studies have reported that individuals with T2DM presented a greater decline of muscle mass and strength compared with euglycemic subjects (10). A recent meta-analysis also demonstrated T2DM patients had a 55% higher risk of sarcopenia compared with those without diabetes (11). In addition, individuals with T2DM are often overweight or obese. Several studies have shown that high body mass index (BMI) has a protective effect on sarcopenia, possibly because high BMI reflects good nutritional status (12, 13). Although BMI can reflect overall nutritional status, BMI cannot differentiate between muscle and fat mass, nor can it reflect fat distribution (14). Some studies have shown that increased body fat was associated with lower muscle mass and muscle quality in general population (15, 16). However, data investigating the effect of fat mass on sarcopenia in patients with T2DM are very limited. To our knowledge, only two studies have investigated the impact of body fat percentage (BF%) on sarcopenia in subjects with T2DM. One study of 87 participants with T2DM has shown BF% derived from dual-energy X-ray absorptiometry(DEXA) is inversely correlated with muscle strength (17). The other study has also suggested that T2DM patients with a high BF% in addition to low BMI might develop sarcopenia (18). However, the above two studies have limitations, such as small sample size and lack of adequate confounders such as nutritional status, insulin resistance, and markers of inflammation. Therefore, understanding the effect and underlying mechanisms of fat mass on sarcopenia in patients with T2DM is crucial to implement appropriate management strategies for sarcopenia.

Therefore, the aim of our study was to assess the effect of BF% on sarcopenia in older adults with T2DM. Additionally, we aimed to explore whether the link between BF% and sarcopenia could be explained by the increased levels of inflammatory indicators and insulin resistance.

## Materials and methods

### Study population

**Figure 1:**
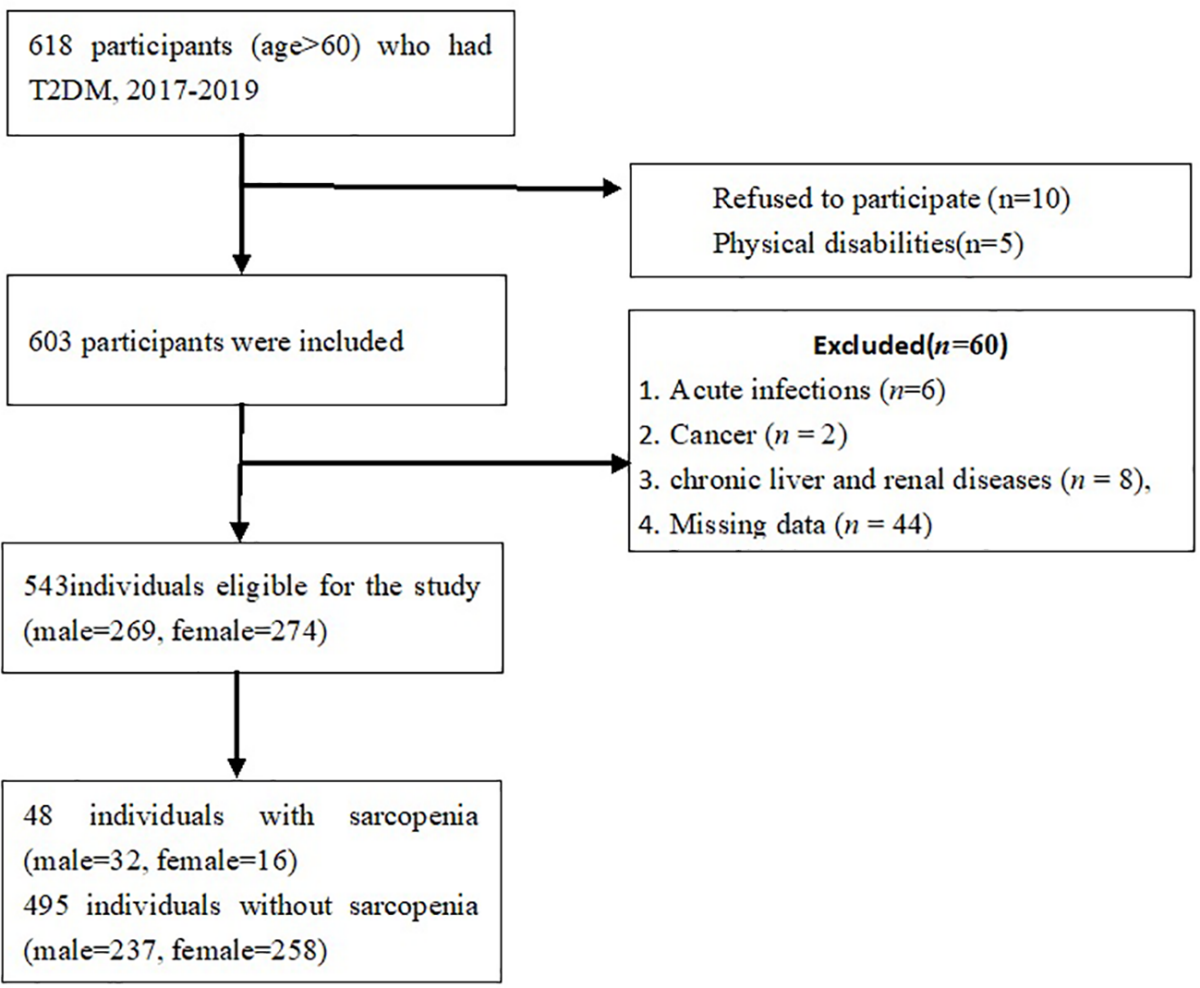
Flow chart on selection of participants.

This was a cross-sectional study of older adult with T2DM, recruited from the wards of Department of Endocrinology, Xuanwu Hospital, Capital Medical University. Participants who were aged > 60 years with a confirmed diagnosis of T2DM were consecutively recruited from Oct 2017 to July 2019. The inclusion criteria were:(1) adults aged over 60 years;(2) met the criteria of the American Diabetes Association for T2DM (19). The exclusion criteria were: (1) the presence of cancer, acute inflammatory diseases, renal and chronic liver diseases;(2) diabetic ketoacidosis and hyperosmolar hyperglycemia; acute myocardial infarction; acute cerebrovascular disease; (3) gastrointestinal diseases such as gastrointestinal bleeding and ulcers, acute and chronic pancreatitis;(4) disability, poor cognitive function. A total of 618 older adults with T2DM were fulfilled the inclusion criteria, among whom, 75 were excluded from this analysis, including 5 subjects with physical disability, 6 with acute infections, 2 with cancers, 8 with renal or liver diseases, 10 refused to participate and 44 with missing data. Thus, a total of 543 subjects(male, n=269; female, n=274)were recruited (**Figure 1**). The *post-hoc* power of the study was estimated using G*power software program (20). The sample sizes revealed > 99% power to detect a significant association (α < 0.05), given an effect size index of 0.1.

The Research Ethics Boards at Xuanwu Hospital of Capital Medical University approved the study protocol (approval number: CTR-IPR-2019002). All participants signed written informed consent.

### Clinical characteristics and biochemical indicators

The participants were interviewed by trained staff. Each participant completed a general questionnaire including demographic variables including age, medical history, and medication records. Height and body weight were measured, and body mass index (BMI) was calculated as weight (kg)/height (m)^2^. The nutritional status of patients was assessed by The Mini Nutrition Assessment (MNA). The sum of the MNA score≥24 were defined as adequate nutritional status, while MNA<24 were defined as malnutrition or at risk of malnutrition (21). Physical activity was assessed by daily exercise: exercise< 0.5 h and exercise≥0.5 h.

Blood samples of the participants were obtained after overnight fasting and were measured in the biochemistry laboratory of Xuanwu Hospital of Capital Medical University. Biochemical parameters, including fasting blood glucose (FBG), hemoglobin A1c (HbA1c), total cholesterol (TCH), triglyceride (TG), high-density lipoprotein cholesterol (HDL), low-density lipoprotein cholesterol (LDL), uric acid (UA), albumin, prealbumin, 25-hydroxyvitamin D_3_ [25(OH)D_3_] and hemoglobin (Hb) were measured. As inflammation indicators, high-sensitivity C-reactive protein (hs-CRP) and interleukin-6 were tested. Fasting blood insulin (FINS), fasting blood C peptide (FCP) were also measured. Insulin resistance (IR) index (HOMA-IR) was calculated by using Homeostasis Model Assessment (HOMA) formula (HOMA-IR = fasting insulin (μIU/mL) × fasting glucose (mmol/L)/22.5).

According to the Asian Working Group for Sarcopenia (AWGS) criteria, individuals with low muscle mass and low muscle strength or low physical performance were defined as having sarcopenia (22).

Body composition data were measured using DEXA (LUNAR iDXA, USA), which measured muscle mass and fat mass for total body, both arms, both legs and the torso. All the scans were performed by a single experienced technologist. Appendicular skeletal muscle (ASM, kg) was determined as the sum of skeletal muscle mass in both arms and legs. Appendicular Skeletal muscle index (ASMI) was defined as ASM divided by height squared (m^2^) (22, 23). Low muscle mass was defined as SMI<7.0 kg/m^2^ for men and < 5.4 kg/m^2^ for women (22). Body fat percentage(BF%) was calculated as total body fat divided by total body mass multiplied by 100. High BF% was defined as BF%≥27% for men or≥40% for women (24).

Muscle strength was defined by handgrip strength using the Jamar^®^ Hydraulic Hand Dynamometer (Patterson Medical, Warrenville, IL, USA). Participants were directed to exert maximum effort three times with each hand. The maximal value was used for further analyses. The cut-off value for low grip strength was set at 28 kg for men and 18kg for women (22).

The physical performance of the participants was measured using usual gait speed (m/s) on a 6- meter course (25). Each participant was instructed to test two times and the faster one was used for defining sarcopenia. The cutoff value for low gait speed was set at <1.0m/s (22).

### Statistical analyses

Statistical analysis was done by using SPSS 21.0. The Kolmogorov-Smirnov test was performed to assess the distribution of the variables. For continuous variables, the data was represented as mean ± SD or median (interquartile range). Categorical variables were reported as frequencies. The Student t-test, Mann-Whitney U test or Chi-square test was used for comparisons between variables where appropriate. The Pearson correlation was utilized to assess the correlations between BF% and ASMI, handgrip strength and gait speed. The independent effects of BF% on sarcopenia were assessed using multiple logistic regression analyses. Mixed effects models adjusting for (1) BF%, age and BMI; (2) BF%, age, BMI, UA, prealbumin, HbA1c, 25(OH)D_3_ were used to evaluate the relationship between BF% and sarcopenia. The results of the regression modeling were presented as odds ratios (ORs) and 95% confidence interval (CI). Two-sided P<0.05 was considered statistically significant.

## Results

### Characteristics of participants

A total of 543 participants with T2DM (67.68 ± 7.06 years; male, n=269; female, n=274) were included in this study. There was no significant difference in age, BMI and duration of diabetes according to sexes (**Table 1**). The biochemical variables of the participants by sex and sarcopenia status were presented in **Table 2**. The overall prevalence of sarcopenia was 8.84%, of which 11.90% for men and 5.84% for women. Compared to the subjects without sarcopenia, those with sarcopenia were significantly older, had lower BMI, higher percentage of exercise <0.5 h and MNA< 24 in both sexes. For men, participants with sarcopenia had higher HbA1c levels, while lower prealbumin, albumin, UA and 25(OH) D_3_ levels. However, no significant difference was found between individuals with and without sarcopenia concerning the biochemical parameters in female participants. There were no significant differences in levels of hs-CRP, interleukin-6, and HOMA-IR and the percentage of usage of antidiabetic drugs between the two groups of either gender (**Table 2**).

**Table 1:** The demographic characteristics of the participants by sexes.

	Total n=543	Male n=269	Female n=274	P value
Age (years)	67.66±6.99	67.33±7.00	67.98±6.97	0.254
Diabetes duration (years)	14.99±8.29	15.49±8.75	14.57±7.90	0.479
BMI (kg/m^2^)	25.66±3.62	25.81±3.51	25.52±3.72	0.335

BMI, body mass index. Data are shown as mean ± standard deviation.

**Table 2:** Characteristics of the participants according to sex and sarcopenia status.

	Male (n=269)			Female (n=274)			
	Sarcopenia n=32	Non-sarcopenia n=237	*P*	Sarcopenia n=16		Non-sarcopenia n=258	*P*
Demographics							
Age (years)	74.01±7.30	66.44±6.54	<0.001		74.19±8.78	67.63±6.77	<0.001
BMI (kg/m^2^)	23.42±3.62	26.07±3.33	<0.001		22.10±3.09	25.75±3.60	<0.001
Lifestyle, n (%)							
Exercise< 0.5 h	13 (40.63)	37 (15.61)	0.001		8 (50.00)	47 (18.22)	0.002
MNA< 24	6 (18.75)	19 (8.02)	0.050		8 (50.00)	37 (14.34)	<0.001
Biochemical markers							
HbA1c (%)	9.20±2.18	8.36±1.77	0.016		9.13±2.45	8.43±1.88	0.165
FBG (mmol/L)	8.44±3.49	9.15±3.44	0.276		9.80±3.88	9.39±3.70	0.668
HOMA-IR	3.33 (1.41-9.11)	4.73 (2.40-8.19)	0.181		5.02 (2.63-9.39	6.00 (3.15-9.91)	0.519
TCH (mmol/L)	4.10±1.03	4.18±1.06	0.667		5.01±0.93	4.57±1.17	0.146
TG (mmol/L)	1.16 (0.96-1.78)	1.33 (0.89-2.28)	0.241		2.00 (1.37-2.60)	1.54 (1.08-2.19)	0.073
HDL (mmol/L)	1.09±0.28	1.13±0.35	0.490		1.53±0.61	1.29±0.36	0.144
LDL (mmol/L)	2.55±0.94	2.54±0.87	0.942		2.74±0.67	2.72±0.95	0.933
UA (mmol/L)	301.63±91.31	334.88±87.97	0.045		343.13±139.14	307.07±80.58	0.101
Prealbumin (mmol/L)	213.25±72.93	257.19±59.74	<0.001		265.63±77.69	241.74±54.53	0.099
Albumin (mmol/L)	38.97±4.29	41.28±4.46	0.006		40.01±3.97	41.64±4.41	0.151
Hemoglobin (g/L)	137.45±16.23	142.55±14.03	0.063		126.13±15.20	128.30±14.27	0.556
25(OH) D_3_(ng/mL)	15.65±8.40	22.63±8.70	<0.001		16.45±6.53	19.36±7.98	0.154
hs-CRP (mg/L)	2.49 (1.55-7.27)	1.99 (1.19-3.38)	0.247		2.75 (1.31-3.57)	2.37 (1.46-4.28)	0.930
Interleukin-6(pg/ml)	5.10 (2.21-9.48)	3.77 (2.42-6.05)	0.089		4.41 (3.07-5.98)	3.71 (5.15-6.30)	0.249
Antidiabetic drugs, n (%)							
Metformin	25 (78.13)	148 (62.45)	0.082		10 (62.50)	188 (72.87)	0.369
Sulfonylurea	10 (31.25)	96 (40.51)	0.315		8 (50.00)	117 (45.35)	0.717
Insulin	19 (59.38)	128 (54.01)	0.567		11 (68.75)	150 (58.14)	0.403
Acarbose	23 (71.88)	164 (69.20)	0.758		13 (81.25)	199 (77.14)	0.941
Thiazolidinedione	9 (28.13)	79 (33.33)	0.556		2 (12.50)	66 (25.58)	0.380
DPP-4 inhibitors	3 (9.38)	8 (3.38)	0.257		1 (6.25)	4 (1.55)	0.261

BMI, body mass index; HbA1c, hemoglobin A1c; FBG, fasting blood glucose; HOMA-IR, homeostasis model assessment of insulin resistance; UA, uric acid; 25(OH)D_3_, 25-OH vitamin D_3_; hs-CRP, high-sensitivity C-reactive protein. MNA, the Mini Nutrition Assessment, MNA<24 was defined as malnutrition or at risk of malnutrition; DPP-4, Dipeptidyl peptidase-4. Data are shown as the mean ± standard deviation or median (interquartile range) or percentage.

### Comparison of body composition and muscle function

A comparison of body composition and muscle function was presented in **Table 3**. As expected, subjects with sarcopenia had lower ASMI, handgrip strength and gait speed for both sexes (P <0.001). For men, BF% was higher in people with sarcopenia compared to those without. However, there was no significant difference in BF% for women.

**Table 3:** Body composition and muscle function according to sex and sarcopenia status.

	Male (n=269)			Female (n=274)		
	Sarcopenia *n*=32	Non-sarcopenia n=237	*P*	Sarcopenia *n*=16	Non-sarcopenia n=258	*P*
ASMI (kg/m^2^)	6.32±0.57	7.78±0.85	<0.001	4.91±0.31	6.47±0.69	<0.001
BF %	32.51±8.79	29.17±5.26	0.043	37.25±6.56	36.79±5.91	0.764
High BF% n (%)	26 (81.25)	154 (64.98)	0.066	5 (31.25)	111 (43.02)	0.355
Handgrip strength(kg)	26.11±6.94	37.11±7.43	<0.001	15.61±3.43	22.50±6.10	<0.001
Gait speed(m/s)	0.82±0.23	1.16±0.24	<0.001	0.67±0.20	1.01±0.29	<0.001

ASMI, appendicular skeletal muscle index; BF%, body fat percentage; High BF% defined as body fat percentage≥27% for males or≥40% for females.

### Correlations between BF% and the components of sarcopenia

**Figure 2:**
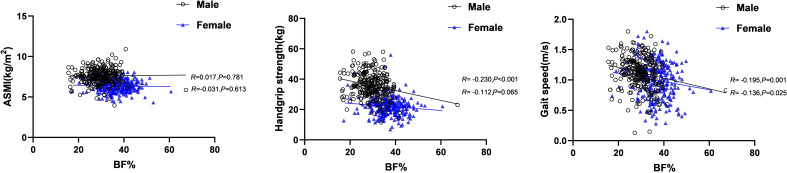
Correlations between BF% and the components of sarcopenia. BF%, body fat percentage.

The Pearson correlation analysis revealed that BF% were negatively correlated with gait speed in men and women (*R* = -0.195, *P* =0.001; *R* = -0.136, *P* =0.025, respectively). For men, BF% was also negatively correlated with handgrip strength (*R* = -0.230, *P* < 0.001). However, no significant association was found between BF% and ASMI of either gender (**Figure 2**).

### Logistic regression models for sarcopenia in both sexes

**Figure 3:**
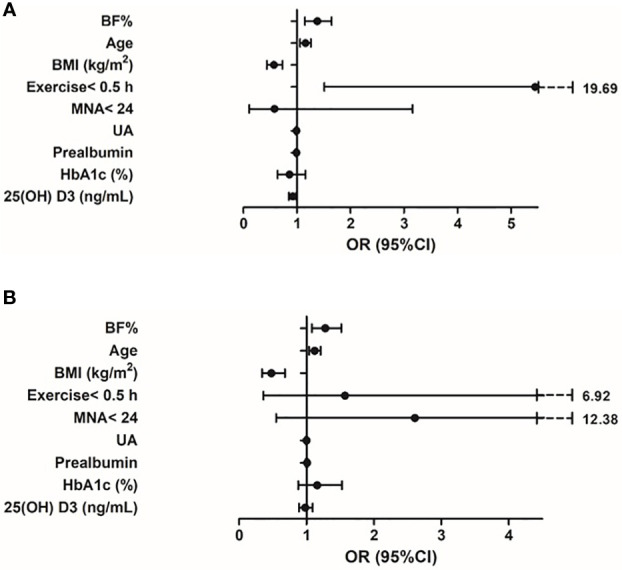
**(A)** Variables associated with sarcopenia in older adults with diabetes (male). Logistic regression model adjusted for BF%, age, BMI, exercise< 0.5 h, MNA<24, UA, prealbumin, HbA1c, 25(OH) D3. **(B)** Variables associated with sarcopenia in older adults with diabetes (female). Logistic regression model adjusted for BF%, age, BMI, exercise< 0.5 h, MNA<24, UA, prealbumin, HbA1c, 25(OH) D_3_.

The logistic regression models for the association between BF% and sarcopenia for men and women were presented in **Table 4A**, **4B**. Age, BMI, exercise< 0.5 h, MNA< 24, UA, prealbumin, HbA1c, 25(OH)D_3_ and BF% were included in the multivariate logistic regression model. In the adjusted model 2, higher BF% was still associated with an increased risk of sarcopenia in both sexes (men, OR: 1.38, 95% CI: 1.15–1.65; women, OR: 1.30, 95% CI: 1.07–1.56), while higher BMI was associated with a decreased risk of sarcopenia (men, OR: 0.57, 95% CI: 0.44–0.73; women, OR: 0.48, 95% CI: 0.33–0.70). In addition, higher UA and 25(OH) D_3_ levels appeared to be protective against sarcopenia in male participants (OR = 0.99, 95% CI: 0.98-1.00; OR = 0.92, 95% CI: 0.85-0.99, respectively), while exercise< 0.5 h was associated with an increased risk of sarcopenia (men, OR: 5.45, 95% CI: 1.51–19.69). However, the other confounders (such as prealbumin, HbA1c) were not related to sarcopenia in both sexes (**Figures 3A, B**).

**Table 4A:** Logistic regression models for risk factors associated with sarcopenia (male).

	Model 1			Model 2		
	Adj. OR	95% CI	*P*	Adj. OR	95% CI	*P*
BF%	1.29	1.14-1.47	<0.001	1.38	1.15-1.65	<0.001
Age	1.16	1.08-1.24	<0.001	1.16	1.06-1.26	0.001
BMI (kg/m^2^)	0.59	0.48-0.71	<0.001	0.57	0.44-0.73	<0.001
Exercise< 0.5 h				5.45	1.51-19.69	0.010
MNA< 24				0.58	0.11-3.16	0.531
UA				0.99	0.98-1.00	0.019
Prealbumin				0.99	0.98-1.00	0.058
HbA1c (%)				0.86	0.64-1.16	0.322
25(OH) D_3_(ng/mL)				0.92	0.85-0.99	0.042

OR, odds ratio; CI, confidence interval; BF%, body fat percentage; BMI, body mass index; MNA, the Mini Nutrition Assessment, MNA<24 was defined as malnutrition or at risk of malnutrition; UA, uric acid; 25(OH)D_3_, 25-OH vitamin D_3_.

Model 1: adjusted with age, BMI.

Model 2: adjusted by Model 1+ exercise< 0.5 h, MNA< 24, UA, prealbumin, HbA1c, 25(OH)D_3_.

The analyses included the following covariates: BF%, age, BMI, exercise< 0.5 h, MNA< 24, UA, prealbumin, HbA1c, 25(OH) D_3_.

**Table 4B:** Logistic regression models for risk factors associated with sarcopenia female.

	Model 1			Model 2		
	Adj. OR	95% CI	*P*	Adj. OR	95% CI	*P*
BF%	1.28	1.08-1.51	0.005	1.30	1.07-1.56	0.007
Age	1.12	1.04-1.21	0.004	1.09	1.00-1.19	0.062
BMI (kg/m^2^)	0.48	0.34-0.68	<0.001	0.48	0.33-0.70	<0.001
Exercise< 0.5 h				1.57	0.36-6.92	0.551
MNA< 24				2.61	0.55-12.38	0.226
UA				1.00	0.99-1.01	0.753
Prealbumin				1.01	0.99-1.02	0.418
HbA1c (%)				1.16	0.88-1.53	0.288
25(OH) D_3_(ng/mL)				0.98	0.89-1.09	0.731

OR, odds ratio; CI, confidence interval; BF%, body fat percentage; BMI, body mass index; MNA, the Mini Nutrition Assessment, MNA<24 was defined as malnutrition or at risk of malnutrition; UA, uric acid; 25(OH)D_3_, 25-OH vitamin D_3_.

Model 1: adjusted with age, BMI.

Model 2: adjusted by Model 1+ exercise< 0.5 h, MNA< 24, UA, prealbumin, HbA1c, 25(OH)D_3_.

The analyses included the following covariates: BF%, age, BMI, exercise< 0.5 h, MNA< 24, UA, prealbumin, HbA1c, 25(OH) D_3_.

## Discussion

In the present study, we assessed the impact of BF% on risk of sarcopenia in a larger group of older adults with T2DM. Our main findings showed that high level of BF% was associated with an increased risk of sarcopenia in both men and women, while higher BMI was protective against sarcopenia, suggesting the importance of assessing BF% instead of assessing BMI alone to manage sarcopenia in T2DM adults.

Sarcopenia has been known as an age-related syndrome and has attracted worldwide attention in recent years. Due to the lack of a single diagnostic criterion, the prevalence of sarcopenia varied considerably with different diagnostic criteria and using different methods applied to measure muscle mass and in different study populations. A recent systematic review and meta-analysis reported the prevalence of sarcopenia ranged from 10%-27% in older adults≥60 years using different classifications and cut-off points (26). Among older Chinese adults, the prevalence was 14% in men and 15% in women based on the AWGS criterion (27). Chung et al. (28) reported that diabetics were associated with a 51% increase in risk for sarcopenia than non-diabetics in a systematic review. Another study in community-dwelling elderly with T2DM, in which sarcopenia was defined using the old AWGS algorithm, reported the prevalence of sarcopenia was 14.8% (29). In our study, the overall prevalence of sarcopenia was 8.84%, which was slightly higher than the prevalence reported by Mori H et al. in a Japanese T2DM population (7.2%) (30), but was lower than that in other populations (31, 32). These differences may be explained by the different diagnostic criteria used and heterogeneous study populations. In our study, DEXA was used to measure muscle mass and the new AWGS algorithm was applied to define sarcopenia.

Additionally, our study suggested that the prevalence of sarcopenia in man (11.90%) was higher than that in women (5.84%). These finding was consistent with the data from Hai et al., which showed the prevalence of sarcopenia was 11% and 9% in community-dwelling men and women aged≥60 years, respectively (33). The decreased levels of insulin-like growth factor 1 (IGF-1) in males and the hormone differences between sexes might be the reason (34). Sex steroid hormones such as testosterone, estrogen and progesterone play important roles in maintaining skeletal muscle quality and function including hypertrophy and the regeneration of damaged muscles (35). Testosterone promotes protein synthesis in muscle, counteracts muscle proteolysis, promotes muscle regeneration, and increases intramuscular insulin-like growth factor-1 (IGF-1) levels (36). Estrogen and progesterone directly exert their effects on muscle tissue through their receptors expressed in skeletal muscle tissue (37). The possible reasons for the higher prevalence of sarcopenia in men than in women are as follows: (1) testosterone levels in healthy men fall by 1% annually from the age of 30 and testosterone deficiency leads to a more robust catabolic response in men than women during aging (38). (2) With aging, the absolute and relative decrease in muscle mass and increases in total fat were more prominent in men than in women (39). In addition, the loss of muscle strength with aging is greater and faster in men (40).

Nutritional status might play a great role on the onset and development of sarcopenia. Several studies have indicated that lower BMI is associated with loss of muscle mass, decline of muscle strength and physical function (41). Older adults are at higher risk of sarcopenia because they are more likely to suffer from malnutrition. Our study also suggested that participants with sarcopenia had higher percentage of MNA< 24 and higher BMI was protective against sarcopenia of either gender. The reason for this finding could be that older adults with higher BMI might have higher protein intake to offset the loss of muscle mass and muscle performance (42). In addition, albumin and prealbumin have been considered as indicators for malnutrition. Higher albumin levels were reported to significantly predict a lower risk of pre-sarcopenia over 4 years in community-dwelling women over the age of 75 (43). One of our previous studies demonstrated that low prealbumin levels are associated with an increased risk of sarcopenia in older men with T2DM (13). In the present study, the levels of albumin and prealbumin were significantly lower in men with sarcopenia compared with those without sarcopenia. However, after adjusting for other confounders, prealbumin levels were no longer associated with the presence of sarcopenia. We also found no association between prealbumin and sarcopenia in older women with T2DM. The inconsistent findings may be due to different confounders applied and the low prevalence of sarcopenia in women (only 16 individuals with sarcopenia). There is growing evidence that sufficient total calorie intake and adequate protein intake, specifically leucine, are important for muscle maintenance (44, 45). Older adults with T2DM may be at additional risk of malnutrition due to excessive dietary restriction to control blood glucose. Thus, ensuring adequate intake of calorie and protein is essential to prevent the development of sarcopenia.

Several previous studies showed that obesity was inversely associated with muscle strength and muscle function in older adults. The data from the Health, Aging, and Body Composition study showed that high fatness was related to lower muscle quality, and it predicted accelerated loss of lean mass (16). Another study also reported that obesity, assessed by BMI, was a risk factor for functional decline in older persons in both sexes (1). However, our study indicated that higher BMI was protective against sarcopenia, which was in line with the findings of several previous studies (46, 47). Although BMI is the most commonly used method to estimate obesity due to the relative ease of application, BMI does not distinguish between fat mass and fat-free mass and may misclassify some older adults (48). Therefore, the reported association of better physical function with higher BMI may be due to the limitation of BMI to define obesity. Thus, BMI was a rough method to evaluate excess body fat, while the body composition measured by DEXA allows us to determine the real fat percentage (49). In the present study, we found BF% were not associated with ASMI, but negatively correlated with gait speed in both sexes, suggesting BF% may be more associated with muscle or physical function rather than muscle mass. Barrett et al. (17) also reported adiposity, assessed as BF% or WC, was inversely associated with muscle strength in community-dwelling older adults with T2DM. The possible explanation for such findings could be that adiposity leads to inter and intramuscular adipose tissue infiltration, which mainly affects muscle function rather than muscle volume. Intramuscular adipose tissue infiltration can induce mitochondrial dysfunction, increase reactive oxygen species formation, and secrete some pro-inflammatory myokines (50). All these mechanisms are involved in the development and progression of sarcopenia. Several studies demonstrated that intramuscular adipose tissue infiltration was associated with decrease in muscle density, loss of muscle quality and physical performance (51, 52). The association between higher BF% and sarcopenia could be explained by increased cytokines levels and insulin resistance. Cytokines secreted by adipose tissue such as interleukin-6, tumor necrosis factor-α may have catabolic effect on muscle (53). In addition, excess adiposity may reduce insulin action, which has pro-catabolic effects on muscle (8). However, we found no significant differences in cytokines and HOMA-IR between the two groups, suggesting that the link between BF% and sarcopenia could not be explained by inflammatory indicators and insulin resistance. This finding coincided with that from Koster et al. (16) who also failed to find the association between adipocytokines, insulin resistance and sarcopenia. The possible explanation for such results could be that the pathophysiological mechanisms linking adiposity to sarcopenia are complex, including hormones, such as testosterone and growth hormone, physical inactivity, etc. Thus, further well-designed studies are needed to investigate the possible mechanisms underlying the link between fat mass and sarcopenia.

Exercise and vitamin D are also important for muscle health. Physical activity and exercise are shown to attenuate age-related decreases in muscle mass, strength, and slow or prevent the development of sarcopenia (54). Our study also showed exercise < 0.5 h was associated with an increased risk of sarcopenia in male participants. Therefore, exercise should be emphasized as part of the lifestyle necessary to maintain muscle health. Previous studies reported that vitamin D levels were positively associated with muscle mass, muscle strength and physical function (55). Our study also indicated that higher levels of vitamin D were protective against sarcopenia in male participants with T2DM. Moreover, *in vivo* studies suggested vitamin D receptors (VDR) overexpression with skeletal muscle hypertrophy. However, mixed findings have been reported for relationships between vitamin D supplementation and sarcopenia indices. A recent systematic review and meta-analysis showed that Vitamin D monotherapy had no effect on any sarcopenia indices in community-dwelling older adults (56). Another meta-analysis suggests that vitamin D plus protein supplementation improves muscle strength in individuals with sarcopenia but has no impact on muscle mass or performance (57). The discrepant findings between studies may be due to heterogeneity between older populations and co-supplementation.

Our study also indicated that uric acid had a protective effect against sarcopenia in older men. Previous studies exploring the associations between uric acid and sarcopenia indices have reported inconsistent results. Beavers et al. (58) demonstrated that low relative skeletal muscle mass was associated with elevated uric acid levels. Allopurinol, a well-known inhibitor of xanthine oxidase (XO), has been reported to play a possible role in the treatment of sarcopenia (59). However, Xu et al. (60), found that higher levels of uric acid were associated with higher muscle mass and muscle strength. Another study showed that higher uric acid levels were linked to better muscle function in the oldest old (61). However, no association between uric acid and sarcopenia was found in women in our study, possibly due to the fewer cases of sarcopenia in females. Since uric acid has both protective antioxidant effect and harmful inflammatory effect, further research is needed to explore its optimal serum levels to maintain good muscle health in older adults.

Although there are several strengths to this study, such as reliable assessments of muscle mass, relatively sufficient confounding factors, certain limitations should be considered. First, causal relationships could not be determined due to the cross-sectional nature of this study. Second, we excluded those individuals with disability and/or poor cognitive function, which may lead to an underestimation of sarcopenia prevalence. Thirdly, our sample was relatively small and was only selected from Xuanwu Hospital, so the results cannot be generalizable to the wider T2DM population. Further well-designed studies with large sample size are needed to assess the impact of BF% on sarcopenia in older adults with T2DM. Fourthly, although the multiple regression analysis was adjusted, some residual confounders such as socio-economic status, comorbidities, and complications were not included in the model. Nonetheless, this study enriches our knowledge of the association between fat mass and sarcopenia.

In conclusion, our results demonstrated that higher BF% was associated with an increased risk of sarcopenia in older adults with T2DM, suggesting the importance of assessing BF% rather than BMI alone to manage sarcopenia. This finding has clinical implications due to the close relationship between sarcopenia and T2DM, suggesting that achieving proper body composition in older adults with T2DM is important for preserving muscle function. Thus, early exercise and dietary interventions aimed at maintaining muscle health might be helpful for preventing sarcopenia in older adults with high BF%.

## Data availability statement

Requests for access to datasets should be directed to xiushuangling@126.com.

## Ethics statement

The studies involving human participants were reviewed and approved by The Research Ethics Boards at Xuanwu Hospital of Capital Medical University approved the study protocol (approval number: CTR-IPR-2019002). All participants signed written informed consent. The patients/participants provided their written informed consent to participate in this study.

## Author contributions

SX and PC were responsible for study conception and design. LS organized the database and drafted the manuscript. JF performed the statistical analysis. LS, XD and ZM summarized the clinical data. All authors contributed to the article and approved the submitted version.
